# Supplementary material for: HIVprotI: an integrated web based platform for prediction and design of HIV proteins inhibitors
Source: J Cheminform. 2018 Mar 9;10:12. doi: 10.1186/s13321-018-0266-y (PMC5845081; doi:10.1186/s13321-018-0266-y)
Supplement: Supplementary file 2 — Additional file 2. Source code of HIVProtI web server. [file 13321_2018_266_MOESM2_ESM.zip › HIVprotI_Source-code/hivprotI/jsmol/cover.htm]

HTML5-only and WebGL Jmol Demo


|  |  |
| --- | --- |
| iPad mode: In this canvas the model is already loading behind the image. It will automatically display when it is loaded. | iPhone mode: In this canvas we just have an image. Clicking on the image will load the code and display the model. |
